# TNFα alters occludin and cerebral endothelial permeability: Role of p38MAPK

**DOI:** 10.1371/journal.pone.0170346

**Published:** 2017-02-07

**Authors:** Yawen Ni, Tao Teng, Runting Li, Agnes Simonyi, Grace Y. Sun, James C. Lee

**Affiliations:** 1 Department of Bioengineering, University of Missouri, Columbia, Missouri, United States of America; 2 Department of Bioengineering, University of Illinois at Chicago, Chicago, Illinois, United States of America; 3 Department of Biochemistry, University of Missouri, Columbia, Missouri, United States of America; University of North Texas, UNITED STATES

## Abstract

Occludin is a key tight junction (TJ) protein in cerebral endothelial cells (CECs) playing an important role in modulating blood-brain barrier (BBB) functions. This protein (65kDa) has been shown to engage in many signaling pathways and phosphorylation by both tyrosine and threonine kinases. Despite yet unknown mechanisms, pro-inflammatory cytokines and endotoxin (lipopolysaccharides, LPS) may alter TJ proteins in CECs and BBB functions. Here we demonstrate the responses of occludin in an immortalized human cerebral endothelial cell line (hCMEC/D3) to stimulation by TNFα (10 ng/mL), IL-1β (10 ng/mL) and LPS (100 ng/mL). Exposing cells to TNFα resulted in a rapid and transient upward band-shift of occludin, suggesting of an increase in phosphorylation. Exposure to IL-1β produced significantly smaller effects and LPS produced almost no effects on occludin band-shift. TNFα also caused transient stimulation of p38MAPK and ERK1/2 in hCMEC/D3 cells, and the occludin band-shift induced by TNFα was suppressed by SB202190, an inhibitor for p38MAPK, and partly by U0126, the MEK1/2-ERK1/2 inhibitor. Cells treated with TNFα and IL-1β but not LPS for 24 h resulted in a significant (p < 0.001) decrease in the expression of occludin, and the decrease could be partially blocked by SB202190, the inhibitor for p38MAPK. Treatment with TNFα also altered cell morphology and enhanced permeability of the CEC layer as measured by the FITC-dextran assay and the trans-endothelial electrical resistances (TEER). However, treatment with SB202190 alone could not effectively reverse the TNFα -induced morphology changes or the enhanced permeability changes. These results suggest that despite effects of TNFα on p38MAPK-mediated occludin phosphorylation and expression, these changes are not sufficient to avert the TNFα-induced alterations on cell morphology and permeability.

## Introduction

Blood–brain barrier (BBB) is a highly selective permeability barrier for protecting the brain from harmful substances circulating in the bloodstream [1]. The neurovascular unit forming the BBB is composed of three major cell types, namely, endothelial cells (ECs), pericytes and astrocytes. ECs are unique as they have continuous intercellular Tight Junction proteins (TJs) and ability to resist immune cells to pass through the BBB and enter into the central nervous system [2]. Many neurodegenerative diseases, such as multiple sclerosis, epilepsy, Alzheimer's disease, or diabetes, show abnormality of TJs function. TJs are comprised of integral membrane proteins such as occludin and claudin, together with the cytoplasmic accessory proteins, such as zonula occluden ZO-1, and ZO-2. Occludin is a major component of the TJ, and is a transmembrane protein present in the plasma membranes of ECs. Its extracellular domains can join one another directly. Occludin is important in maintaining TJ stability and BBB function. Immunoblotting and immunocytochemistry show distribution of occludin continuously at cell-cell contacts in brain ECs [3].

Tight junction proteins such as occludin are highly regulated by multiple signaling pathways and are phosphorylated by different protein kinases. Mitogen-activated protein kinases (MAPKs) represent a highly conserved family of Ser/Thr protein kinases (ERK, p38/MAPK, and JNK), which are involved in a variety of fundamental cellular processes, such as proliferation, differentiation, apoptosis, and survival [4]. There is evidence linking phosphorylation of occludin and the paracellular permeability of ECs. Hyperpermeability of ECs is associated with dephosphorylation of occludin at Thr residues and hyperphosphorylation at the Tyr site [5]. Other studies showed that phosphorylation of specific Tyr residues in occludin may regulate its interactions with ZO-1 and other TJ proteins [6].

Under physiological setting, the BBB may be affected by its environment including exposure to microbiome and concomitant altering of cellular immune responses. The microbial by-products, such as LPS, and the pro-inflammatory cytokines such as TNFα and IL-1β, can cause irreversible damage to the TJs and alter BBB functions. Thus, it is important to uncover the underlining mechanisms of how these pro-inflammatory factors modulating TJ molecules.

In this study, we investigate effects of TNFα, IL-1β and LPS on occludin expression in the human endothelial cells (hCMEC/D3) and relate their effects to intercellular permeability function. Our study demonstrated ability for TNFα to stimulate MAPKs and the involvement of phospho-ERK1/2 and phospho-p38MAPK to elicit transient phosphorylation of occludin. Prolonged exposure of TNFα to these cells also caused a decrease in occludin expression, changes in cell morphology, and altered permeability functions. However, despite blocking partially of the decreased occludin expression by p38MAPK inhibitor, this kinase action is not sufficient to ameliorate TNFα -induced changes in morphology and permeability functions.

## Materials and methods

### Cell culture

The Human Cerebral Microvascular Endothelial Cell Line (hCMEC/D3) was obtained from Cellutions Biosystems (CLU512, Ontario, Canada) and maintained at complete EBM-2 medium at 37°C in 5% CO_2_. Complete medium (final concentration) EBM-2: EBM-2 Endothelial basal medium (Lonza, #190860, Basel, Switzerland), 5% Fetal Bovine Serum (Life Technologies, #14190, Carlsbad, CA); 1% Penicillin-Streptomycin (Life Technologies, #15140–122); 1.4 μM Hydrocortisone (Sigma, #H0135, St. Louis, MO); 5 μg/mL Ascorbic acid (Sigma, #A4544); 1/100 Chemically Defined Lipid Concentrate (Life Technologies, # 11905031); 10 mM HEPES (Life Technologies, #15630–080); 1 ng/mL bFGF (Sigma, #F0291).

Inserts and flasks/Petri dishes were pre-covered with rat Collagen I lower viscosity (R&D Systems, #3443-100-01, Minneapolis, MN). For coating, rat collagen was diluted in 0.02M acetic acid to a final protein concentration of 5 μg/mL. Sufficient amount of solution was added to cover the surface of culture dish and incubate at 37°C for 1 h. This was followed by washing with PBS (Life Technologies, #1653508) three times and then replaced with culture medium. Cells were passed twice weekly and seeded on Petri dishes or flasks at a density of 25,000 cells per cm^2^. Three-four days after seeding on flasks or Petri dishes, cells reached confluence and can be trypsinized and used until passage 35.

### Western blotting

Confluent hCMEC/D3 cells were serum starved with EBM-2 Endothelial basal medium for 3 h. Inhibitors SB202190 (Cell Signaling, #8158S, Danvers, MA), U0126 (Cell Signaling, #9903S) were used at concentration of 10 μM, and were added to cells 15 min prior to adding TNFα (10 ng/mL) (ThermoFisher, #PHC3015, Waltham, MA), IL-1β (10 ng/mL)(R&D Systems, #AD1414092) or LPS (100 ng/mL) (Sigma, #L6893). Cells were incubated at different times, and were washed with PBS once, and ready for further analysis.

For Western blotting, hCMEC/D3 cells were washed with PBS and lysed in 300 μL RIPA buffer (10 mM Tris-Cl (pH 8.0), 1 mM EDTA, 0.5 mM EGTA, 1% Triton X-100, 0.1% sodium deoxycholate, 0.1% SDS, 140 mM NaCl) (Abcam, Cambridge, UK) per dish on ice for 15 min. Cells were scraped from dish to Eppendorf tube, and centrifuged at 100,000 × g for 1 min. The supernatants were mixed with 2x Laemmli buffer supplemented with 10% β-mercaptoethanol (ThermoFisher, #35602) and heated for 5 min. Protein concentration was determined using a Pierce^™^ BCA Protein Assay Kit (ThermoFisher, #23225). Equivalent amounts of protein (10 μg) for each sample were resolved in SDS-polyacrylamide gels for electrophoresis, running at 110V for 120 min. For occludin, ERK1/2 and β-actin, 7.5% SDS-polyacrylamide gels were used, and for p38MAPK, 10% SDS-polyacrylamide gels were used.

After electrophoresis, gels were overlaid with methanol-activated polyvinylidene difluoride (PVDF) membrane (BioRad, Hercules, CA) under constant voltage 100 V for 100 min. PVDF membrane was then blocked with 5% BSA (M/V) (Sigma,#SLBD7265V) for 1h in room temperature and followed by incubation with the primary antibody (1:1000) overnight at 4°C. Primary antibodies for occludin was from ThermoFisher (OC-3F10), and p-P44/42 MAPK (T202/Y204) (4377S), p44/42 MAPK (ERK 1/2,) (9107S), p-p38 MAPK (T180/Y182) (9211S), and p38 MAPK (9212S) were from Cell Signaling. HRP-conjugated secondary antibody: Anti-rabbit IgG (Cell Signaling, 7074s) and anti-mouse IgG (Cell Signaling, 7076s) was diluted in 5% milk at 1:5000 ratio. HRP-anti-occludin (331520) was purchased from Life Technologies, and HRP-conjugated β-actin was from Sigma (#111M4793). Unbound proteins were washed off by 0.05% (v/v) Tween 20 in TBS (Bioexpress, #0283C285, Kaysville, UT). Protein expression level was examined by using enhanced chemifluorescence (ThermoFisher) and the laser scanner LAS-3000 (FujiFilm, Tokyo, Japan) for exposure. If necessary, stripping buffer (ThermoFisher, #21059) was used to remove bound antibodies at room temperature for 20 min, and samples were reblotted the same way as indicated above. Western blotting band intensity was determined using the Quantity One software (Bio-Rad).

### Co-immunoprecipitation

After treatment protocol, hCMEC/D3 were washed with PBS and lysed with RIPA buffer (50 mM Tris-Cl, pH 7.4, 150 mM NaCl, 0.5% sodium deoxycholate, 0.1% SDS, and 1% NP-40, Boston Bioproducts, BP-115) together with phosphatase /protease inhibitor cocktail (Cell Signaling, 5872S) for 20 min. After mixing, 10 μL Protein A beads (Santa Cruz, CA) with 5 μg P-Thr (H-2) (Santa Cruz, sc-5267) or P-Tyr (PY99) antibody (Santa Cruz, sc-7020) for 2 h, 200 μL lysates were diluted with 1xPBS buffer to 1 mL, and followed by addition of the pre-mixed antibody-protein A mixture. After overnight incubation at 4°C, beads were washed with 1x PBS for 3 times and centrifuged at 8000 rpm, 2 min at 4°C. Finally, beads were suspended in 60 μL 1x PBS, and SDS-loading buffer was added to denature the protein sample. The precipitated proteins were resolved by Western blot.

### Assessment of cell morphology

For study to examine cell morphology, cells were cultured in 35 mm dish. After serum starved for 3 h and pretreatment with inhibitors for 15 min, cells were stimulated with TNFα, IL-β and LPS for 24 h. Cells were examined using a phase contrast Nikon DIAPHOT 300 microscope attached with a CCD cool camera and linked to the MagnaFire 2.1C software for image processing. Normally, 3–4 bright field pictures of cells were captured from each dish. Cell dimension (width vs length) was measured using the Image J 1.48v software program. Typically, nine cells from each picture were randomly selected for measurement. Experiments were repeated with cells grown in three different passages.

### Dextran assay for measurement of cell permeability

**Fig 1:**
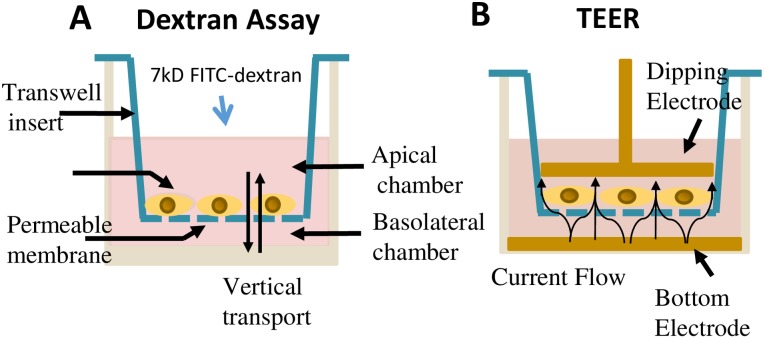
Schematic description of transendothelial permeability assays. (A) Diagram depicting method for Dextran assay protocol. (B) Diagram depicting the measurement using the TEER protocol.

hCMEC/D3 cells were cultured on 24-well plate with 3.0 μm pore size transwell inserts (Fisher Scientific, Corning Inc., 353096) for 7days (until confluent). Each insert was washed with 1x HBSS once, and transferred to fresh 24-well plate (Fig 1A). Then, 200 μL of HBSS containing 1μg/mL FITC-Dextran was added to the upper chamber and 600 μL of HBSS to the lower chamber (Fig 1A). The 24-well plate with transwell inserts was incubated for 1 h at 37°C at 5% CO_2_ with slight shaking. The concentration of FITC-Dextran transferred to the lower chamber was determined using the Microplate Reader (Biotek, 258632, Winooski, VT) with excitation and emission wavelengths of 492 nm and 520 nm, respectively.

### Measurement of transendothelial electrical resistance (TEER)

In this study, endothelial barrier function was assessed using the TEER protocol (Fig 1B). hCMEC/D3 cells were cultured on 12-well plate with 3.0 μm pore size transwell inserts for 7days (until confluent). An endothelial volt/ohm meter for TEER-EVOM2 (World Precision Instruments, Sarasota, FL) was used to measure the TEER value. The TEER values were obtained by transferring the transwell inserts into the Endohm chamber (Fig 1B). The concentric pairs of electrodes above and beneath the membrane caused a coincident current density flow across the membrane, and EVOM2 offered the transmembrane electrical resistance according to the current. All TEER values were determined after subtracting the background and timing of insert membrane.

### Cell viability assay

Cell viability was measured using the WST-1 assay protocol. Briefly, hCMEC/D3 cells were cultured in 96-well plate at a density of 25,000 cells per cm^2^. After 3 days, culture medium was replaced with EBM-2 Endothelial basal for 3 h. Cells were then washed with PBS, and then incubated with 25 μL/well WST-1 (Sigma, #5015944001) for 1 h. After incubation and shaking, the formazan dye formed was quantitated using the microplate reader at 570 nm. Optical density values were determined after subtracting the background.

### Data analysis

Data are shown as mean ± SD from at least three independent experiments. Statistical analyses were carried out using either one-way or two-way analysis of variance (ANOVA) followed by Bonferroni post-test. Differences were considered significant at p<0.05.

## Results

### TNFα, IL-1β but not LPS induced rapid and transient band-shift in occludin

Pro-inflammatory cytokines such as TNFα and IL-1β and endotoxins such as LPS have been shown to stimulate oxidative and inflammatory responses in immune active cells and alter cell functions. Since TJs are important for endothelial cell functions, the goal for this study is to investigate whether cytokines and endotoxins alter occludin expression and function in the hCMEC/D3 cells. In this study, cells were treated with or without TNFα (10 ng/mL), IL-1β (10 ng/mL) and LPS (100 ng/mL) for 15, 30 min or 1, 2, 4, 6 hours. Cell lysates were collected and occludin expression pattern was analyzed by immunoblotting assay. Initially, we observed a thin band-shift of occludin upon incubation of cells with TNFα and followed by resolution with 10% gel. However, we found that the two bands were better resolved upon using 7.5% gel. Therefore, in subsequent experiments, 7.5% gel was used for blotting occludin and ERK1/2, and 10% gel was used for blotting p38MAPK.

**Fig 2:**
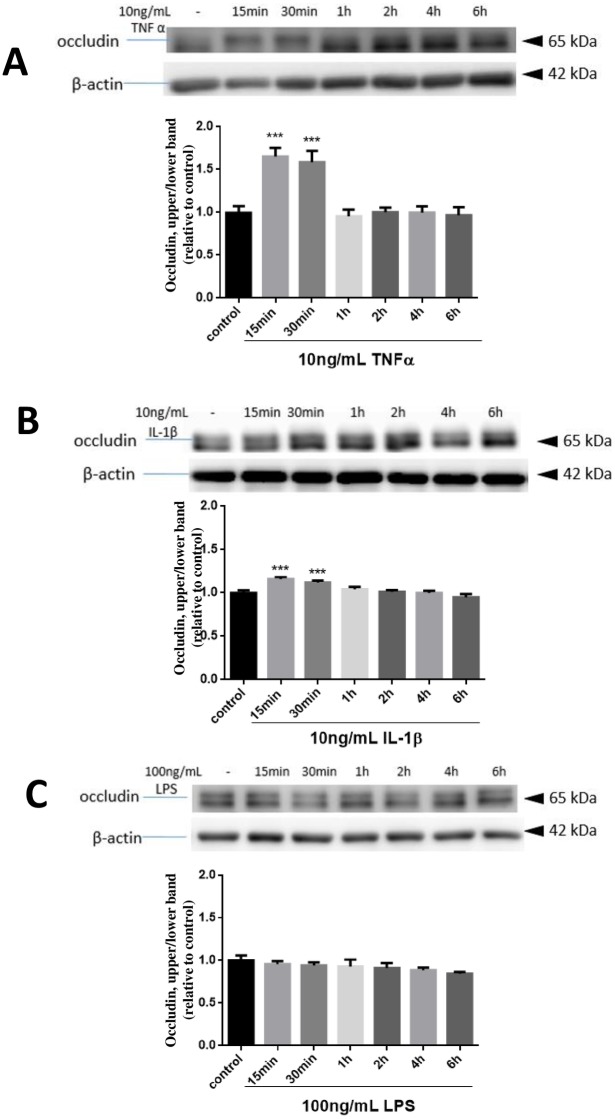
Effects of TNFα, IL-1β and LPS on occludin band-shift in hCMEC/D3 cells. Cells were treated with or without TNFα (10 ng/mL) (A), IL-1β (10 ng/mL)(B) and LPS (100 ng/mL) (C) for 15, 30 min and 1, 2, 4, 6 hours. Cell lysates were collected and occludin and β-actin expression patterns were analyzed by immunoblotting assay as described in text. The density of upper band versus total upper and lower and normalized with β-actin was determined by measuring protein intensity as PIupper/PItotal/PIβ-actin. Results are mean ± SD from 4 or more experiments and data are analyzed by one-way ANOVA followed by Bonferroni post-tests (***P<0.001 compared with no treatment control).

In this study, TNFα exposure triggered a transient band-shift for occludin noticeable at 15 and 30 min (Fig 2A). This shift from the lower band to upper band readily returned to normal within 1 hour. Quantification of the proportion of upper band versus total upper and lower bands revealed significant increase of 65.2% at 15 min and 59.3% at 30 min in TNFα -treated cells as compared with the control cells (Fig 2A). Treatment with IL-1β also resulted in a band-shift at 15 and 30 min (Fig 2B), but the extent of changes were much smaller as compared with TNFα, displaying only 15.8% and 11.9% increase in 15 and 30 min, respectively. In contrast, addition of LPS (100 ng/mL) did not show apparent band-shift (Fig 2C). Further testing with high levels of LPS up to 10 μg/mL for 2 h did not show transient occludin band-shift (S1 Fig).

### TNFα and IL-1β but not LPS mediated phosphorylation of ERK1/2 and p38MAPK in hCMEC/D3 cells

**Fig 3:**
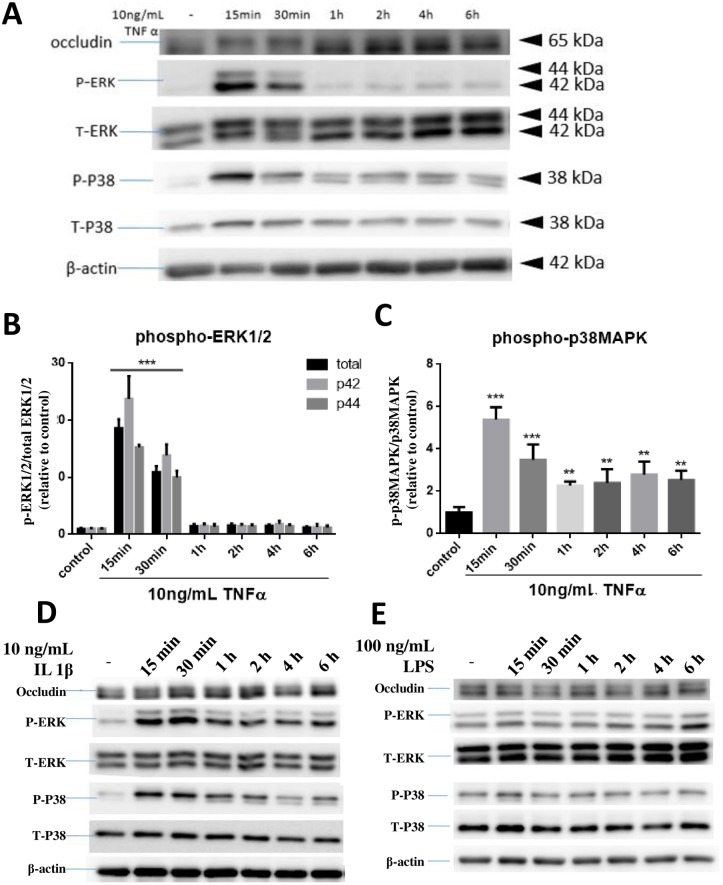
Effects of TNFα, IL-1β and LPS on p-ERK1/2 and p-p38MAPK expression in hCMEC/D3 cells. Cells were treated with or without TNFα (A, B, C), IL-1β (D) and LPS (E) for 15, 30 min and 1, 2, 4, 6 hours. Cell lysates were collected and phospho-ERK1/2 (P- ERK), total ERK1/2 (T-ERK), phospho-p38MAPK (P-P38), total p38MAPK (T-P38) and β-actin expression pattern were analyzed by immunoblotting assay. Quantification of phospho-proteins was determined through assay of PIphospho/PItotal/PIβ-actin and then normalized to control. Results of (B) and (C) are mean ± SD from 4 or more experiments and data are analyzed by one-way ANOVA followed by Bonferroni post-tests **P<0.01, ***P<0.001 compared with no treatment control). Results of (D) and (E) are representation of two repeated experiments.

We hypothesized that the observed band-shift for occludin is due to post translational modifications such as phosphorylation. Since these cytokines have been shown to activate the MAPK pathways [7], an experiment was carried out to examine the time course for TNFα and IL-1β to stimulate ERK1/2 and p38MAPK in hCMEC/D3 cells. As shown in Fig 3A, treatment of hCMEC/D3 cells with TNFα led to a transient increase in phosphorylation of ERK1/2 (p-ERK1/2) and p38MAPK (p-p38MAPK) as early as 15 min, and this event correlated well with the time for occludin band-shift. In this experiment, both p42 and p44 p-ERK showed similar activation pattern (Fig 3A and 3B). TNFα-induced increase in p-p38MAPK also peaked at 15 min but levels remained slightly above control levels even after 6 hours (Fig 3A–3C).

Treatment of cells with IL-1β at 10 ng/mL also caused transient increases in p-ERK1/2 and p-p38MAPK at 15–30 min (Fig 3D), and with increasing time, the levels gradually decreased but were maintained at slightly higher levels than non-treated controls up to 6 hours (Fig 3D). Similar to occludin, no transient changes in levels of p-ERK1/2 and p-p38MAPK were observed with LPS (100 ng/mL) (Fig 3E). As with occludin, testing with higher levels of LPS also did not result in transient increase in phosphorylation of ERK1/2 and p38MAPK (S1 Fig). Taken together, these results suggest a possible correlation between the occludin band-shift and phosphorylation of ERK1/2 and p38MAPK in hCMEC/D3 cells.

### TNFα-induced occludin band-shift showed a better temporal relationship with phosphorylation of p38MAPK than with ERK1/2

**Fig 4:**
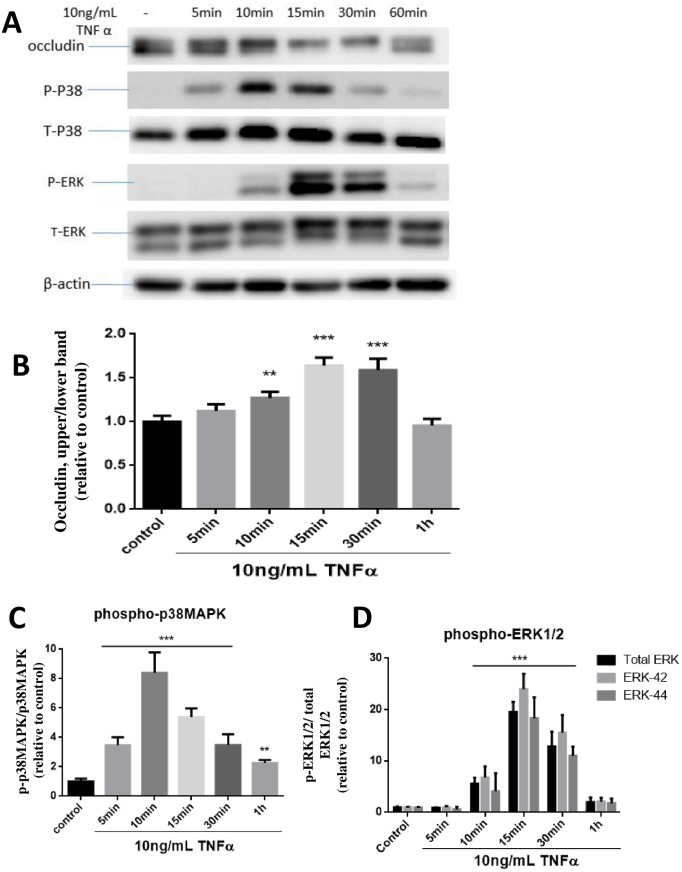
TNFα-mediated occludin band-shift and phosphorylation of ERK1/2 and p38MAPK in hCMEC/D3 cells. Cells were treated with or without TNFα (10 ng/mL) at 5, 10, 15, 30, 60 min (A). Cell lysates were collected and occludin, P- ERK, T-ERK, P-P38, T-P38 and β-actin expression pattern was analyzed by immunoblotting assay. Quantification of the proportion of occludin upper band were determined through PI_upper_/PI_total_/PI_β-actin_ and then normalized to control (B). phospho-ERK1/2 (C) and phospho-p38MAPK (D) were similarly quantified and plotted. Results are mean ± SD from 3 or more experiments and data are analyzed by one-way ANOVA followed by Bonferroni post-tests (**P<0.01, ***P<0.001 compared with no treatment control).

In order to better pinpoint the signaling events and occludin band-shift at early time points, cells were stimulated with TNFα and phosphorylation of p38MAPK and ERK1/2 were investigated at 5, 10, 15, 30 and 60 min after TNFα treatment. In this experiment, occludin band-shift could be detected as early as 10 min after TNFα exposure, but reached a maximum at 15 and 30 min, and returned to basal level at 1h (Fig 4A and 4B). TNFα-induced increase in p-p38MAPK was observed as early as 5 min, peaked at 10 min and then declined (Fig 4A–4C). Meanwhile, the increase in p-ERK1/2 was not detected until 10 min and peaked at 15 min prior to the decline (Fig 4A–4D). Based on these time course data, TNFα-induced occludin band-shift appeared to follow a better temporal relationship with p-p38MAPK than with p-ERK1/2.

### Inhibition of p-ERK1/2 and p-p38MAPK reduced occludin band-shift

**Fig 5:**
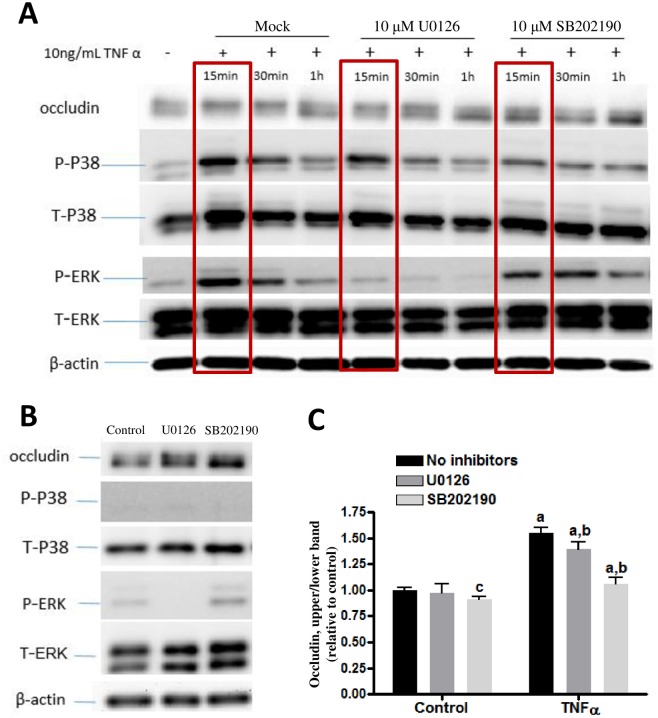
Effects of p-ERK1/2 and p-p38MAPK inhibitors on occludin band-shift in CMEC/D3 cells. Cells were pretreated with or without 10 μM U0126 and 10 μM SB202190 for 15 min and then stimulated with or without 10 ng/mL TNFα for 15, 30, 60 min (A and C). (B) Testing the effects of inhibitors alone. Cell lysates were collected and occludin, P- ERK, T-ERK, P-p38, T-p38 and β-actin expression pattern was analyzed by immunoblotting assay. Quantification of the proportions of occludin upper band at 15 min of TNFα and with inhibitors (blots enclosed in brackets) were determined through PI_upper_ /PI_total_/PI_β-actin_ and then normalized to control. Results are mean ± SD from 4 or more experiments. Two-way ANOVA revealed significant main effects of TNFα and the inhibitors and a significant interaction (p<0.0001 for each). Bonferroni post-test showed significant differences between TNFα-treated groups as compared to their respective controls (as indicated by the letter “a”), SB202190 alone as compared to control/no inhibitors (as indicated by the letter “c”) and TNFα-treated groups with vs. without the inhibitors (as indicated by the letter “b”).

In order to further determine whether TNFα-induced phosphorylation of ERK1/2 and p38MAPK is involved in occludin band-shift, specific inhibitors were used, i.e., U0126 for MEK1/2-ERK1/2 and SB202190 for p-p38MAPK. In this experiment, hCEMEC/D3 cells were incubated with the respective inhibitors for 15 min prior to stimulation with TNFα for 0, 15, 30, and 60 min. As indicated before, occludin band-shift was observed when cells were treated with TNFα for 15 and 30 min and returned to control levels by 1 h (Fig 5A). Incubation with inhibitors alone did not alter occludin phosphorylation (Fig 5B). Pre-incubation of the MEK1/2 inhibitor, U0126 (10 μM), for 15 min followed by stimulation of TNFα almost completely prevented the increase in p-ERK1/2 (Fig 5A, Lane 5–7), indicating that the inhibitor is functional. However, preincubation with the p-p38MAPK inhibitor, SB202190 (10 μM), only partially inhibited p-p38MAPK induced by TNFα (Fig 5A, Lane 8–10). Nevertheless, SB202190 was able to block the TNFα-induced occludin band-shift almost completely, whereas U0126 only blocked the TNFα-induced band-shift by 24% (Fig 5C). Additionally, SB202190 alone slightly reduced occludin band-shift in controls (Fig 5C). Together, these results show that suppression of p-p38MAPK and partially p-ERK1/2 could block the TNFα-induced occludin band-shift, and suggest a critical role for p38MAPK in phosphorylation of occludin by TNFα.

In the above study, we observed that SB202109 at 10μM only partially decreased p-p38MAPK expression. Subsequent information about SB202190 revealed that it inhibits multiple forms of p38MAPK. Apparently, SB202190 was able to inhibit at least one specific form of p38MAPK and this form of p38MAPK was the active form for phosphorylation of occludin. Taken together, these results demonstrate an effective link between occludin band-shift and induction of p-p38MAPK by TNFα.

### Immunoprecipitation to assess tyrosine and threonine phosphorylation of occludin

**Fig 6:**
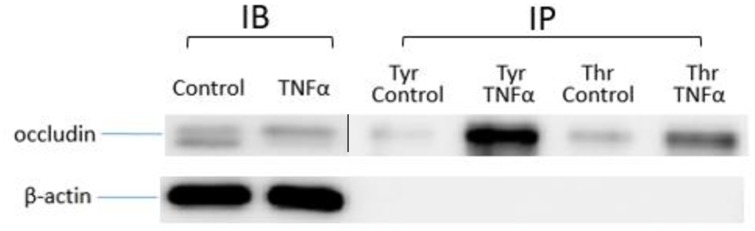
Effects of TNFα on tyrosine and threonine phosphorylation of occludin. hCMEC/D3 cells were treated with or without 10 ng/mL TNFα for 15 min. Cell lysates were mixed with anti-tyrosine or anti-threonine antibody conjugated with protein A beads and then phosphorylated proteins in cell lysates were pulled down. Occludin and β-actin expression pattern was analyzed by immunoblotting assay.

Since a number of kinase pathways and different kinases have been shown to phosphorylate occludin, we performed immunoprecipitation assay to test phosphorylation at the tyrosine and threonine residues. In this study, anti-tyrosine or anti-threonine antibodies conjugated with protein A beads were used to pull down phosphorylated proteins in cell lysates after treatment with and without TNFα. Occludin in the cell lysates were subsequently detected by Western blot. As shown in Fig 6, exposure of cells to TNFα for 15 min increased the upper band intensity of occludin in total cell lysates. After immunoprecipitation, occludin appeared only in the upper band regardless of with or without TNFα exposure, confirming that the upper band corresponded to the phosphorylated occludin. Under this condition, both anti-tyrosine and anti-threonine antibody interacted with and pulled down phosphorylated occludin (p-occludin), suggesting that occludin can be phosphorylated by either type of kinases. In fact, phosphorylation by tyrosine kinases appeared to be more prominent than threonine kinases.

### Long-term exposure to TNFα and IL-1β but not LPS decreased occludin expression

**Fig 7:**
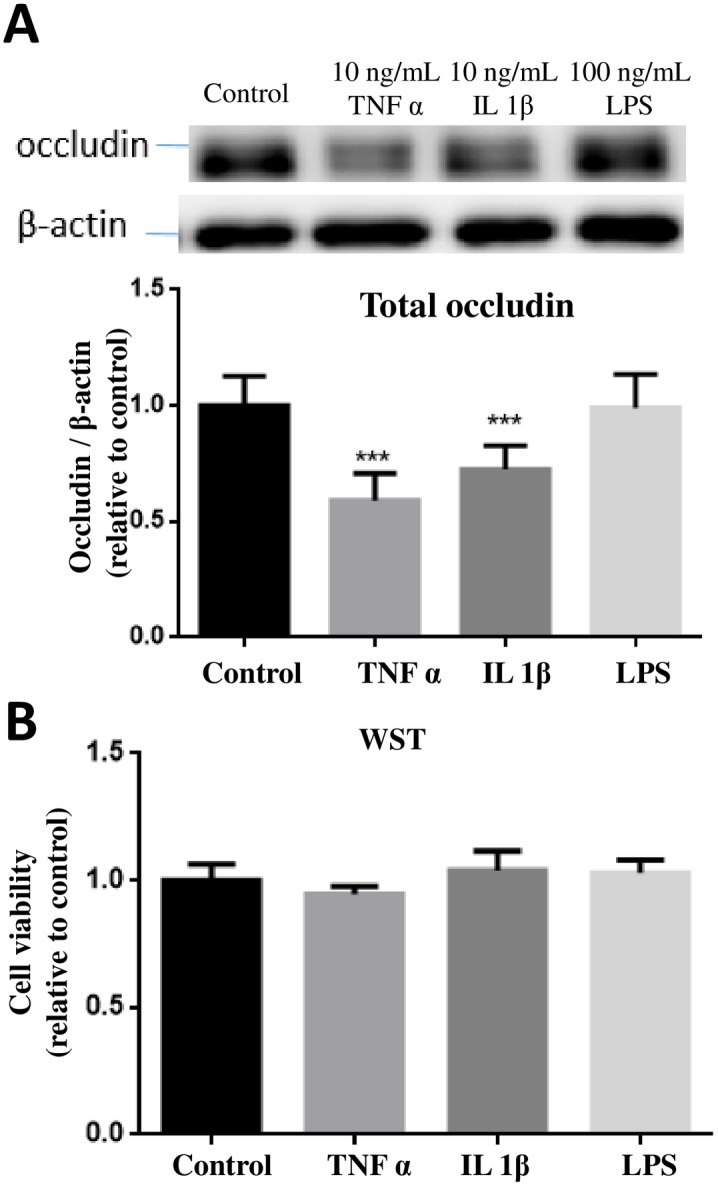
Effects of TNFα, IL-1β and LPS on occludin expression and cell viability. (A) hCMEC/D3 cells were treated with or without TNFα (10 ng/mL), IL-1β (10 ng/mL) and LPS (100 ng/mL) for 24 hours. Cell lysates were collected and occludin and β-actin expression pattern was analyzed by immunoblotting assay. Quantification of the protein band intensity was determined through PI_total_/PI_β-actin_ and then normalized to control. Results are mean ± SD from 4 or more experiments and data are analyzed by one-way ANOVA followed by Bonferroni post-tests (***P<0.001 compared with no treatment control). (B) Cell viability was determined by the WST-1 assay. One-way ANOVA revealed no significant differences among the groups.

Studies so far indicated ability for TNFα to induce phosphorylation of p38MAPK and ERK1/2 and a temporal relationship between increased p-p38MAPK and occludin band-shift. Since a major function for occludin is to mediate TJ activity among endothelial cells which may further lead to regulation of BBB, studies were carried out to test whether the early signaling changes induced by TNFα may lead to subsequent physiological and morphological changes of endothelial cells. In this study, hCMEC/D3 cells were exposed to TNFα (10 ng/mL), IL-1β (10 ng/mL) and LPS (100 ng/mL) for 24 hours and changes in occludin expression as well as cell viability were determined. As shown in Fig 7A, TNFα induced a significant decrease in occludin expression (40.9%) as compared with the control group. To a lesser extent, treatment of cells with IL-1β also reduced occludin expression (27.53%), whereas treatment of LPS did not show apparent changes on occludin expression (Fig 7A). None of these conditions alter cell viability (Fig 7B).

**Fig 8:**
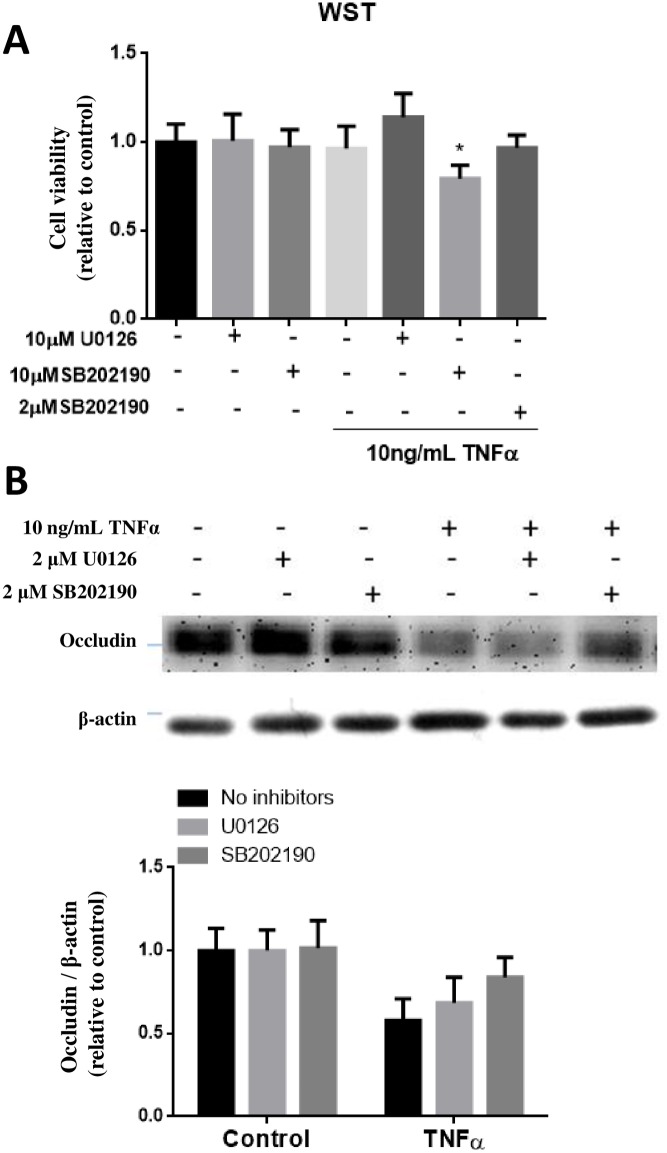
Effects of p-ERK1/2 and p-p38MAPK inhibitors on TNFα-induced occludin expression in hCMEC/D3 cells. (A) Initial testing for cell viability using WST-1 assay indicated toxicity of cells upon incubation (24h) with TNFα (10 ng/mL) in the presence of SB202190 at 10 μM but not at 2 μM. (B) Testing ability of U0126 (2 μM) and SB202190 (2 μM) to ameliorate the decrease in occludin expression upon exposure of TNFα for 24 h. Cell lysates were collected and occludin and β-actin expression pattern was analyzed by immunoblotting assay. Quantification of protein band intensity was determined through PItotal/PIβ-actin and then normalized to control. Results are mean ± SD from 5 or more experiments. Two-way ANOVA revealed a significant main effect of TNFα (p<0.0001) and the inhibitors (p = 0.0287). Bonferroni post-test showed significant differences between TNFα-treated groups as compared to their respective controls (as indicated by the letter “a”), and TNFα-treated groups with vs. without SB202190 (as indicated by the letter “b”).

Since exposure of hCMEC/D3 cells to TNFα and IL-1β resulted in the decrease in expression of occludin, study was carried out to test whether the decrease in occludin expression could be blocked by the MAPKs inhibitors. Initially, exposure of cells to 10 μM of SB202190 for 24 h indicated slight decrease in cell viability, and this problem was resolved by reducing SB202190 to 2 μM (Fig 8A). Using the 2 μM concentration, pretreatment with SB202190 was able to significantly (p<0.01) prevent the decrease in occludin expression due to TNFα, whereas no reversal effect was observed with 2 μM of U0126 (Fig 8B).

### TNFα-induced changes in hCMEC/D3 cell morphology

**Fig 9:**
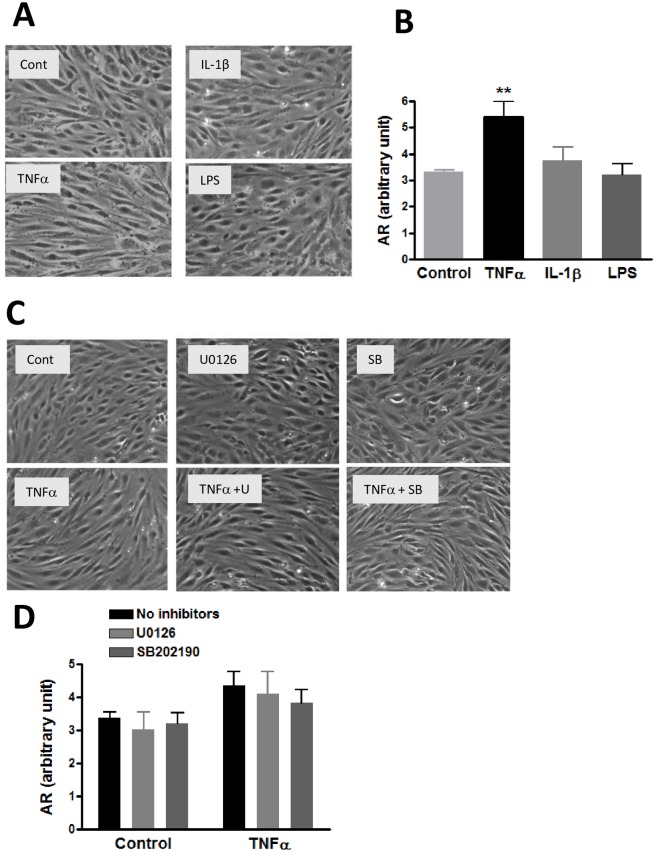
Effects of TNFα, IL-1β and LPS on morphology of hCMEC/D3 cells. (A) Cells were treated with or without TNFα (10 ng/mL), IL-1β (10 ng/mL) and LPS (100 ng/mL) for 24 hours and observed under bright field microscope as described in text. Representative pictures were taken from different areas in the field. (B) Ten cells from each picture were randomly selected for measurement using the Image J protocol. Results are mean ± SD from 3 experiments. One-way ANOVA with Bonferroni post-test showed a significant difference (p<0.01) between control and TNFα. (C) Representative bright field photomicroscope pictures to assess effects of MEK1/2 and p-p38MAPK inhibitors on TNFα-induced morphological changes. Cells were pretreated with U0126 (2 μM) and SB202190 (2 μM) for 15 min prior to treatment with TNFα (10 ng/mL) for 24 h. (D) Results are analyzed as in (B). Data are expressed as the mean ± SD of three experiments. Two-way ANOVA showed a significant main effect of TNFα (p = 0.0013). The effects of the inhibitors were not significant.

Exposure of cells to cytokines and LPS could also result in changes in cell morphology. Bright field microscopic imaging showed that control hCMEC/D3 cells are irregular and star-like shape whereas TNFα-treated cells showed a spindle-like shape with more narrow and elongated morphology (Fig 9A). Estimation of the relative cell area using the Image J program indicated only TNFα but neither IL-1β nor LPS could induce changes in cell morphology (Fig 9B). In a subsequent study, we further tested whether TNFα-induced morphological changes is associated with activation of MAPKs and phosphorylation of occludin. Cells were preincubated with inhibitors for p-ERK1/2 (2 μM U0126) and p-p38MAPK (2 μM SB202190) and subsequently stimulated with TNFα for 24 h. Results indicated neither inhibitors could prevent changes in cell morphology induced by TNFα (Fig 9B).

### Long-term exposure to TNFα, IL-1β and LPS differentially enhanced endothelial permeability

**Fig 10:**
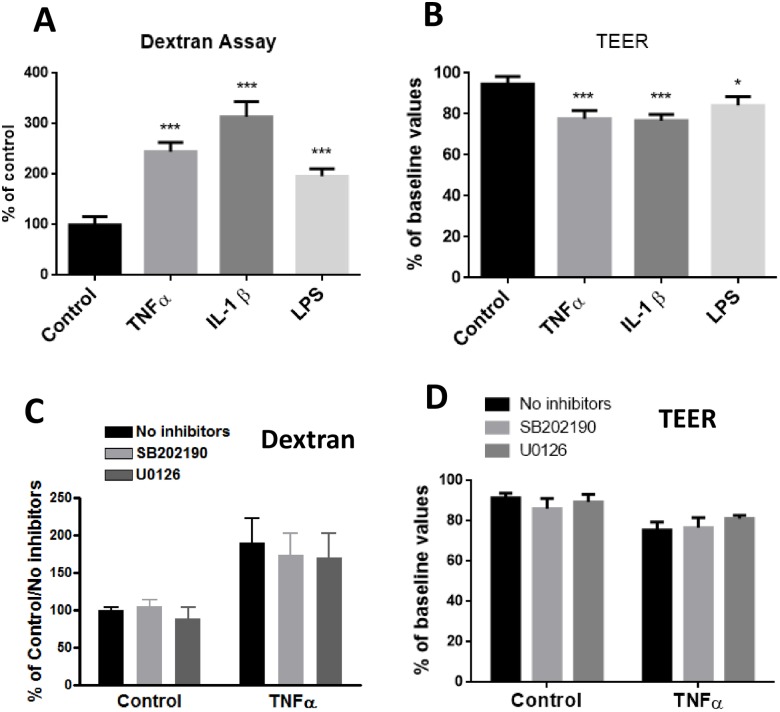
Effects of TNFα, IL-1β and LPS on paracellular permeability as measured by the Dextran and TEER assays. (A) For the Dextran assay, cells were cultured in inserts for 24 h followed by applying fluorescent FITC-Dextran beads as described in Methods. (B) TEER was determined using an endothelial volt/ohm meter for TEER-EVOM2 as described in Methods. Permeability were determined through FI24h/FI0min (Fluorescence Intensity) and then normalized to control. Results are mean ± SD from 4 or more experiments and data are analyzed by one-way ANOVA followed by Bonferroni post-tests (*P<0.05, ***P<0.001 compared with no treatment control). (C and D) Assessing effects of p-ERK1/2 and p-p38MAPK inhibitors on TNFα-induced changes on paracellular permeability as measured by the Dextran (C) and TEER assays (D). Data are expressed as the mean ± SD of four or more experiments. The results were analyzed by two-way ANOVA, and a significant main effect of TNFα was revealed (p<0.0001 for each). The effects of the inhibitors were not significant.

Using the Dextran and TEER assay protocols, we attempted to measure paracellular permeability in hCMEC/D3 cells upon treatment with TNFα and MAPK inhibitors. Enhanced endothelial permeability is frequently marked by a decrease in TEER and increase in transfer of Dextran across the cells. After 24 hours treatment of cells with TNFα (10 ng/mL), IL-1β (10 ng/mL) and LPS (100 ng/mL), paracellular permeability measured by exposing cells to 7 kD FITC-Dextran indicated an increase in ability for Dextran to transfer across the cells with potency ranking of IL-1β>TNFα >LPS (Fig 10A). Under similar conditions, TEER measurement due to TNFα was reduced by 22.27%, IL-1β by 23.21%, and LPS by 15.85% (Fig 10B).

Subsequent study was carried out to test whether inhibition of MAPKs could block the permeability changes induced by TNFα. As shown in Fig 10C and 10D, pretreatment of cells with MEK1/2 and p38MAPK inhibitors did not reverse the permeability changes induced by TNFα. Taken together, these results indicated ability for TNFα to decrease occludin expression, change in cell morphology and increase in permeability, the link between occludin expression and p38MAPK is not sufficient to explain the changes in cell morphology and permeability function.

## Discussion

### TNFα induces transient phosphorylation of occludin in hCMEC/D3 endothelial cells

In this study, we demonstrated effects of TNFα to cause an upward band-shift of occludin in hCMEC/D3 endothelial cells. This band-shift was noticeable as early as 5 min, and reaching a maximum around 15 min before returning to basal level within 1 hour. The band-shift is regarded as increase in molecular weight of the compound, most likely due to phosphorylation. Indeed, several studies have demonstrated phosphorylation for occludin by different protein kinases in different cell systems [8, 9]. However, studies to link specific kinase to function of this molecule have been limited due to the multiple phosphorylation sites. In this study, we observed an upward band shift upon stimulation of hCMEC/D3 cells by TNFα. In MDCK cells, occludin was resolved into several bands between 62 and 82 kD in SDS-PAGE, and these bands were converged into the lowest molecular weight band by alkaline phosphatase treatment, a protocol known to remove the phosphate groups (10). Phosphoamino acid analyses indicated that the higher occludin bands were comprised of phosphoserine and phosphothreonine residues [10]. Similar to our study, injecting VEGF into the vitreous cavity of rat eye caused a modification of occludin in the retina, with a band-shift from 60 to 62 kDa by 15 min post-injection and reaching a maximum at 45 min [11]. Phosphorylation and dephosphorylation are known to play essential roles in regulating cell metabolism, such as regulation of protein-protein interaction, protein degradation, and enzyme activity. The transient nature of band-shift after stimulation with TNFα in hCMEC/D3 cells suggests the presence of phosphatases for dephosphorylation of this molecule. Results here are in agreement with the notion showing the importance of kinases and phosphatases in the regulation of occludin in endothelial cells.

### TNFα-induced occludin band-shift is correlated with increase in MAPK

Mitogen-activated protein kinases (MAPKs) are involved in a variety of fundamental cellular processes, such as proliferation, differentiation, apoptosis, and survival [4]. Previous studies have demonstrated activation of MAPKs pathways by pro-inflammatory cytokines and LPS in endothelial cells. For example, TNFα induced a rapid ERK1/2, p38MAPK, JNK activation in human umbilical vein endothelial cells [12]. Similar to our study, cells in endothelium-derived permanent human cell line (EA.hy926) also showed increase in phosphorylation of p38MAPK by TNFα within 5 min and quickly dephosphorylated within 30 min [13]. In our study, exposure of TNFα (10 ng/mL) to hCMEC/D3 cells induced transient increase in phosphorylation of ERK1/2 and p38MAPK with a slightly different time course. While TNFα-induced p-p38MAPK increase was noticed as early as 5 min and rapidly declined after 10 min, the increase in p-ERK1/2 was not detected until 10 min and peaked at 15 min prior to the decline. Under the same conditions, occludin band-shift became obvious at 5 min after TNFα exposure, and reached a maximum at 15 min. Taken together, the time profile for TNFα to stimulate occludin band-shift appears to show a better relationship with the increased phosphorylation of p38MAPK than ERK1/2.

Besides band-shift by TNFα, IL-1β at10 ng/mL also caused a small band-shift of occludin and this cytokine also transiently increased in the expression of p-ERK1/2 and p-p38MAPK at 15–30 min. On the other hand, LPS at100 ng/mL elicited no obvious changes in occludin band-shift and this level of LPS also did not cause transient increase in p-pERK1/2 and p-p38MAPK (Fig 2). Experiment to test higher levels of LPS (up to 10 μg/mL) also did not show obvious band-shift and increase in MAPKs (S1 Fig). In a recent study by Qing et al., high concentrations of LPS at 10 μg/mL was shown to enhance p38MAPK and JNK phosphorylation in hCMEC/D3 cells [14]. Our study was originally based on the levels of LPS used with BV-2 microglial cells, where LPS at 100 ng/mL could induce a rapid increase in phosphorylated p38MAPK and ERK 1/2 [15]. Taken together, these data suggest that endothelial cells, at least the hCMEC/D3 cells, are less responsive to LPS as compared to microglial cells.

In order to further link TNFα-induced phosphorylation of p38MAPK and ERK1/2 with occludin band-shift, inhibitors for these MAPKs were applied. In this study, p-ERK1/2 expression was completely abrogated by pretreatment with U0126, the inhibitor for MEK1/2 that phosphorylates ERK1/2, indicating potent effect of this inhibitor (Fig 5A). Interestingly, pretreatment of SB202190 (10 μM), inhibitor for p38MAPK, only partially inhibited p-p38MAPK expression upon stimulation with TNFα. It became obvious that there are four p38 MAP kinases in mammals: α, β, γ and δ, also known as stress-activated kinase 2a (SAPK2a), SAPK2b, SAPK3 and SAPK4, respectively [16]. Study by Davies et al [17] demonstrated that SB202190 only inhibit p38α and p38β, whereas p38γ and p38δ were completely unaffected by this compound [17]. Therefore, the incomplete inhibition of p-p38MAPK by SB202190 can be explained by presence of other isoforms of p38MAPK in our cells. Taken together, our data are consistent with results suggesting presence of p38MAPK isoforms in mediating TNFα-induced occludin band-shift.

### Long time exposure of TNFα and IL-1β decreased occludin expression

A number of studies, including those in our laboratory, observed a decrease in total occludin expression after treating endothelial cells with TNFα for 24h [18]. In our study, exposure of IL-1β but not LPS also caused the decrease in occludin (Fig 7A). Occludin expression was decreased in human endothelial cells HUVECs upon exposure to TNFα [19]. Study by Cui et al. [20] observed a reduction of occludin mRNA levels in intestinal epithelial cells of mice at 6 h after TNFα injection, suggesting of possible transcriptional regulation [20]. Besides mRNA, occludin expression can be regulated by other mechanisms, e.g., post-translational mechanisms such as ubiquitination, phosphorylation/dephosphorylation and/or degradation by extracellular proteases. TNFα is known to activate nuclear factor kappa-light-chain-enhancer of activated B cells (NF-κB), a transcription factor, involved in inflammatory responses. In our study, TNFα-induced decrease in occludin expression could be reverted to a great extent by SB202190, the p38MAPK inhibitor, further linking the role for p38MAPK in regulation of occludin biosynthesis. In the study by Boivin et al., inhibition of NF-κB prevented the increase of T84 intestinal epithelial TJ permeability and depletion of occludin induced by IFNγ [21]. However, how alteration of the NF-κB pathway led to down-regulation of occludin and subsequent TJ disruption in endothelial cells is still not clear and thus is an important area worthy of further investigation.

### Long time TNFα treatment altered endothelial morphology and cell barrier functions

Results in this study show that upon exposure of TNFα (10 ng/mL), IL-1β (10 ng/mL) and LPS (100 ng/mL) to hCMEC/D3 cells for 24 h, morphologic changes were observed only in cells treated with TNFα (Fig 9). On the other hand, TNFα, IL-1β and LPS were able to cause significant increases in paracellular permeability as indicated by the Dextran and TEER assays (Fig 10). Using the same cell line and treated with 1, 10 and 100 ng/mL TNFα, study by Lopez-Ramirez et al. [22] also observed a significant increase in paracellular permeability to 70 kD FITC-dextran at 24h upon treatment with10 ng/mL TNFα [22]. The mechanism for LPS to increase permeability change is not well understood, since LPS did not cause morphologic change. Nevertheless, study by Banks et al. indicated decrease in TEER at low ng levels of LPS added to primary brain endothelial cells [23]. In our study, neither the inhibitors for ERK1/2 or p38 MAPK could reverse changes in paracellular permeability induced by TNFα (Fig 10). Results from our study indicate that alteration of occludin is not sufficient to explain the changes in cellular physical properties due to exposure to TNFα. Obviously, more studies are needed to better understand the complex pathways and activities of other TJ proteins.

## Supporting information

S1 Fig Effects of LPS doses on occludin band-shift and time-dependent increase in phosphorylation of ERK1/2 and p38MAPK. hCBEC/D3 cells were treated with 100 ng/mL, 1μg/mL and 10 μg/mL of LPS and incubated for 15, 30 min and 1, 2 h. Cell lysates were subjected to Western blot analysis for occludin, p-ERK1/2 and total ERK1/2, p38MAPK and p-38MAPK, and β-actin.
(TIF)
