# Supplementary figures and images for: TNFα alters occludin and cerebral endothelial permeability: Role of p38MAPK
Source: PLoS One. 2017 Feb 7;12(2):e0170346. doi: 10.1371/journal.pone.0170346 (PMC5295672; doi:10.1371/journal.pone.0170346)

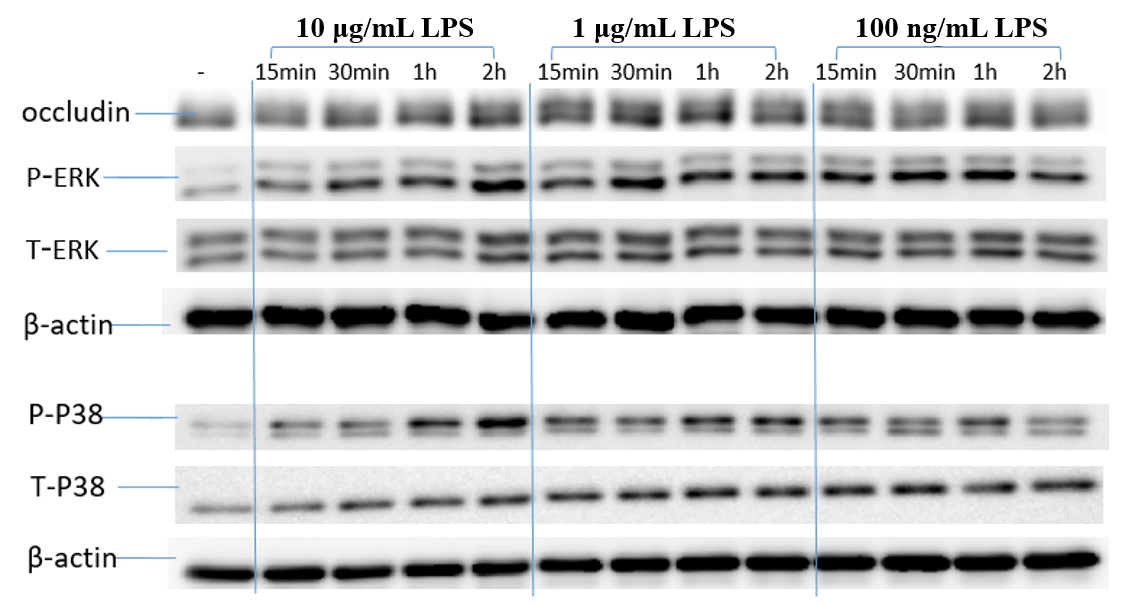

Supplement: S1 Fig — hCBEC/D3 cells were treated with 100 ng/mL, 1μg/mL and 10 μg/mL of LPS and incubated for 15, 30 min and 1, 2 h. Cell lysates were subjected to Western blot analysis for occludin, p-ERK1/2 and total ERK1/2, p38MAPK and p-38MAPK, and β-actin. (TIF) [file pone.0170346.s001.tif]
